# Assessing Student Attitudes Regarding Cost-Consciousness in Medical Education

**DOI:** 10.15694/mep.2019.000012.1

**Published:** 2019-01-11

**Authors:** Marisa Leon-Carlyle, Rory McQuillan, Ioana Baiu, Amy Sullivan, Dmitry Dukhovny, Neel Shah

**Affiliations:** 1 University of Toronto; 2 Stanford University; 3 Harvard Medical School; 4 Oregon Health & Science University

**Keywords:** medical education, resource stewardship, learner values, Choosing Wisely, Costs of Care

## Abstract

This article was migrated. The article was marked as recommended.

**Purpose**: The purpose of this study was to compare attitudes regarding cost-consciousness between student populations at two medical schools in the United States and Canada.

**Method**: We conducted a cross-sectional survey of students at Harvard Medical School and University of Toronto. We performed chi-square analyses comparing responses from the two institutions.

**Results**: Response rates were 48% (n=162) and 45% (n=228) at Harvard and the University of Toronto, respectively. At both institutions, >96% of students agreed clinicians at all stages of training should be familiar with cost-conscious decision-making, 80% agreed physicians are responsible for discussing healthcare costs with patients, and over 80% felt they had too little education on the topic in medical school. Students differed in opinions about the extent to which patients should inquire about costs, with students at Harvard more likely to endorse this opinion compared with those from Toronto (51% vs 28%, respectively), and differed over whether cost-consciousness led to rationing of healthcare (Harvard 30% vs Toronto 51%). Fewer than 10% of all students expressed concerns that incorporating costs into care was unethical. Overall, 85% of students from both countries would like more formal teaching on this topic.

**Discussion**: Students from both schools strongly endorsed a need to learn more about cost-conscious decision-making. Findings suggest students in both systems can benefit from learning similar core concepts related to high-value, cost-conscious care, and teaching in this topic can be customized to reflect specific differences in expectations and practices in the two healthcare systems.

## Introduction

The unsustainable growth of healthcare expenditures has motivated national conversation about areas of potential waste in United States healthcare delivery. ( Truffer *et al.*, 2010) A recent Institute of Medicine report estimated that more than $760 billion-roughly 30% of total U.S. healthcare costs-are spent on tests and procedures that do not lead to health improvements. ( Yong and Olsen, 2010) Although there is debate about how much of this amount is under the direct control of physicians, some estimate that physicians may determine up to 80% of healthcare expenditures. ( Crosson, 2009) As a result, there have been calls for reducing overtreatment and overutilization of tests and procedures as a means of containing costs. ( Berwick and Hackbarth, 2012) The American Board of Internal Medicine (ABIM) Foundation’s Choosing Wisely Campaign has brought considerable traction to this effort through a partnership of more than fifty medical specialties that each identified five commonly used tests or procedures that physicians and patients should question. ( *Costs of Care*, 2013)

Conversion to a single-payer healthcare system similar to the Canadian system is often proposed as one potential solution. The Canadian healthcare system is primarily publicly-funded by a single payer - the provincial government. Nonetheless, Canadian healthcare faces similar challenges with ballooning healthcare costs. This is particularly of concern given the limited financial resources of provincial governments. For example, in Ontario, 42% of public expenditures are devoted to healthcare, and this is predicted to rise to 70% in the next 12 years. ( Government of Ontario, 2012) Concerns about unsustainable healthcare spending and patient safety led to the launch of a Choosing Wisely Canada campaign. This campaign has attained similar success partnering with over sixty Canadian medical organizations to release 160 stewardship recommendations since 2014. ( *Choosing Wisely Canada*, 2013) Included in these recommendations is a list of six things medical students and trainees should question developed by the Canadian Federation of Medical Students (for example, “Don’t hesitate to ask for clarification on tests, treatments, or procedures that you believe may be ordered inappropriately”). ( Lakhani *et al.*, 2016)

In both countries, the provision of cost-conscious care has been discussed as a parallel solution in medical education. ( Weinberger, 2011) Currently, our teaching culture rewards thoroughness while disincentivizing restraint and uncertainty. ( Detsky and Verma, 2012; Simpkin and Schwartzstein, 2016) During morbidity and mortality conferences sins of omission are discussed at length while sins of commission are discussed rarely. Indeed, medical students are often encouraged and even graded based on their ability to present long lists of differential diagnoses, emphasizing the breadth of knowledge and not necessarily the thoughtfulness in making high-value decisions. ( Rosenbaum and Lamas, 2012) As a first step toward understanding similarities and differences between these two countries in student attitudes and educational needs related to cost-conscious care, we compared responses from surveys of medical students at Harvard Medical School and the University of Toronto. Findings can inform the development and use of curricula that can serve the needs and interests of students in these two different healthcare systems.

### Introduction

Since 1979, several studies have shown that medical students, residents, and even experienced senior physicians have limited knowledge of healthcare costs for medical tests and procedures. ( Dresnick, Roth and Linn, 1979; Graham, Potyk and Raimi, 2010) A thirteen-year old survey of first- and fourth-year U.S. medical students tested familiarity with health policy and attitudes towards their medical curriculum and found significant knowledge gaps and an overall perception that the current medical curriculum falls short of addressing these issues. ( Agrawal *et al.*, 2005) A more recent study unveiled inconsistency in cost-conscious practices among internal medicine residents that suggest a need for curricula that emphasize stewardship of medical resources. ( Green, Bell and Wenger, 2013)

Some argue that cost-consciousness should be taught as “a positive professional value” in order to support resource stewardship practices for future generations of physicians. ( Cooke, 2010) As part of the 2011 Accreditation Council for Graduate Medical Education (ACGME) six general competencies, the “Systems-Based Practice” category requires physicians in training to “incorporate considerations of cost awareness and risk-benefit analysis in patient and/or population-based care as appropriate.” ( Accreditation Council for Graduate Medical Education, 2011) Steven Weinberger, President of the American College of Physicians, proposed to emphasize the importance of costs by creating a 7 ^th^ ACGME competency dedicated solely to the cost-conscious care and stewardship of resources. ( Weinberger, 2011) The Medicare Payment Advisory Commission (MedPAC) proposed changes to graduate medical education (GME) funding using pay-for-performance financial incentives “to support people within the GME system who know that we need to do a better job in preparing physicians for the healthcare delivery system of tomorrow”. ( Iglehart, 2010) In Canada, the CanMEDS 2015 Framework lists the abilities to “allocate healthcare resources for optimal patient care” and to “apply evidence and management processes to achieve cost appropriate care” as enabling competencies that all medical curricula should address. ( Frank *et al.*, 2015)

In recent years, there have been several initiatives to develop curricula at the GME level, including the *Costs of Care Teaching Value Project* and the American College of Physician’s *High Value, Cost-Conscious Care Initiative.* ( *Costs of Care*, 2013) Short formal teaching sessions have been proven effective at increasing medical students’ and/or residents’ knowledge and attitudes of cost-conscious decision-making. ( Manheim *et al.*, 1990; Aagaard *et al.*, 2010; Sommers *et al.*, 2012) Some medical schools have started incorporating the topic of high-value care and cost-conscious decision-making. ( Smith, 2012; Leon-Carlyle and Srivastava, 2015) Nonetheless, a gap in teaching cost-conscious care persists, particularly at the medical school level. ( Cooke, 2010) A prior survey of U.S. medical students found that students agree physicians have an ability to contain costs, but also perceive barriers to cost-conscious care in the role-modeling in the clinical environment. (Hunderfund *et al.*, 2017) There remains limited data regarding medical students’ attitudes desire to learn more about these topics.

The purpose of this study was to fill this data gap by examining medical students’ attitudes regarding healthcare costs. In particular, we were interested in assessing whether there were any notable differences between students studying in a public, single-payer (the University of Toronto) versus a private, multi-payer healthcare system (Harvard Medical School). The hypothesis was that students from a publicly funded healthcare system might be more aware of healthcare system costs due to pressures from public funding, as opposed to the private multi-payer system where students may be shielded from this knowledge.

## Methods

### Study population

The study population consisted of second- and fourth-year medical students at Harvard Medical School and the University of Toronto. We chose these two populations to reflect the level of training at the end of the pre-clinical and clinical years of medical school training, respectively.

### Survey instrument

We developed a 25-item electronic survey instrument using the Qualtrics ^©^ (Qualtrics, Provo, Utah) survey software platform to assess knowledge and attitudes regarding the role of cost-consciousness in medical education. ( *Qualtrics*, 2013) The domains covered by the survey included: 1) demographic information, 2) formal and informal training in costs of care, 3) responsibility for cost-conscious decision making, 4) the role of cost-consciousness in specific types of clinical scenarios, 5) sources of cost-conscious knowledge, 6) attitudes regarding costs of care and cost-conscious decision making, 7) cost-conscious curriculum at their medical school. We hypothesized that attitudes among medical students may be influenced by stage of training, professional goals and prior experiences. With this hypothesis in mind, the survey was designed with de novo questions based on literature review and consultation with expert medical educators. Pre-testing was performed to ensure clarity and comprehensibility. A pilot survey was administered to a separate group of 15 students with a background in medicine and public health; the questionnaire was refined based on these preliminary results and suggestions.

The format of the survey includes multiple-choice, Likert scale questions, True/False items, and free text entry. Students’ attitudes related to costs of care were tested using Likert-type agree/disagree response scales, and attitudes towards their medical curriculum were tested using multiple-choice, Likert-type questions. At the end of the survey we provided an opportunity for free-text entry of comments and suggestions to better incorporate cost-conscious decision-making into the undergraduate medical curriculum. An electronic copy of the survey is available by request.

### Survey implementation

At Harvard Medical School, the survey and reminder e-mails were distributed via e-mail through the Qualtrics platform. Participation in the study was voluntary and responses were confidential. A Frequently Asked Question (FAQ) sheet was provided along with the recruitment e-mails and was available as a reference on the survey website. The study was open for six weeks and weekly reminders were sent to those students who had not completed the questionnaire. The study received approval of the Harvard Medical School Committee on Human Subjects.

At the University of Toronto, a paper-survey was distributed during mandatory large-group lectures to second- and fourth-year students. These paper surveys were gathered and entered in Microsoft Excel by a research assistant. Participation in the study was both anonymous and voluntary. The study received approval of the University of Toronto Research Ethics Board.

The questionnaire was sent to a total of 333 medical students at Harvard Medical School (n=169 second-year students and n=164 fourth-year students) and distributed to 512 students at the University of Toronto (n=256 second-year students and n=256 fourth-year students).

### Statistical analyses

Survey answers were grouped into categories according to positive, neutral, or negative valence. For example, ‘Strongly Agree’ and ‘Agree’ responses were analyzed as ‘Agree.’ The same was done for ‘Strongly Disagree’ and ‘Disagree,’ while ‘Neutral’ responses were kept as an independent group. The data were analyzed using STATA ^©^ and SAS ^©^ 9.4 statistical software. For items containing ordinal answer choices (e.g., the Likert-scale answer choices), we utilized a chi-square analysis to detect differences between two groups. For open-ended questions, we carried out a content analysis to identify major themes.

A priori, we explored the differences in responses based on location of the medical school. Eighty-two percent of surveys were filled out completely; the remaining 18% had minimal missing data, with an average of 2.9 items missing. We used all available data and did not impute for missing data.

## Results/Analysis

### Study sample

The response rate was 48% (n=162) with an equal distribution of gender and year in school for Harvard Medical School. The response rate was 45% (n=228) with 60% females and 40% males responding, and with 66% second year students and 34% fourth year students responding at the University of Toronto. Table 1 illustrates the demographics of our study population. 44 (27%) and 17 (7%) of the Harvard and the University of Toronto medical students, respectively, had completed prior degrees including Masters in Public Health, Business Administration, Education or other degrees.

**Table 1.** Sample characteristics

Characteristic	Harvard Medical School n=162	University of Toronto n=228	*p ^a^*
**Age** **<26** **26-30** **31-40**	70 83 9	110 93 18	.16
**Gender** **Male** **Female**	88 74	136 91	.01
**Year of Study** **2nd** **4th**	84 78	151 77	.01
**Prior Studies** **MPH** **MBA** **MSEd** **PhD** **Other ^b^**	10 10 28 14	4 1 0 1 11	.03 .001 .10 .004 .10

Abbreviations: MPH Masters of Public Health, MBA Masters of Business Administration, MSEd Masters of Science in Education, PhD Doctor of Philosophy

^a^ p-value compares Harvard Medical School vs. University of Toronto

^b^ Other includes: DMD Doctor of Medicine in Dentistry, MPP Masters of Public Policy, MA Masters of Arts, MS Masters of Science, MEd Masters in Education, ScD Doctor of Science, MPhil Masters of Philosophy

### Attitudes regarding costs of care and curricular changes

Regardless of place of study, nearly all respondents believed that clinical students (96%), residents (98%), and attendings (99%) should be familiar with costs of care and cost-conscious decision-making. Just over half of U.S. students (54%) and nearly two-thirds of Canadian students (65%) thought that first- and second-year students should also be familiar with cost-conscious decision making ( *p* < 0.05) (see Table 2).

Eighty-one percent of Harvard Medical School and University of Toronto medical students agreed that physicians are responsible for discussing costs of care with their patients. School differences were found for opinions about whether patients were also responsible for inquiring about the costs of care, with 51% of Harvard Medical School students agreeing with this statement compared with 12% of University of Toronto students who believe patients are also responsible ( *p* < 0.0001) (see Table 2).

**Table 2.** Attitudes towards cost-conscious healthcare responsibility

	Combined	Harvard Medical School	University of Toronto	*p ^a^*
**1st and 2nd year medical students should be familiar with costs of care and cost-conscious decision-making.** **Agree** **Neutral** **Disagree**	n=389 60.6% 32.1% 7.7%	n=162 53.7% 41.3% 4.9%	n=227 64.8% 25.6% 9.7%	.002
**3rd and 4th year medical students should be familiar with costs of care and cost-conscious decision-making.** **Agree** **Neutral** **Disagree**	n=389 96.4% 3.1% 0.5%	n=162 96.2% 3.1% 0.6%	n=227 96.5% 3.1% 0.4%	.97
**Residents should be familiar with costs of care and cost-conscious. decision-making** **Agree** **Neutral** **Disagree**	n=339 98.2% 1.5% 0.3%	n=162 98.1% 1.2% 0.6%	n=177 98.3% 1.7% 0.0%	.54
**All attendings, regardless of specialty, should be familiar with costs of care and cost-conscious decision-making.** **Agree** **Neutral** **Disagree**	n=387 98.7% 1.0% 0.2%	n=161 99.3% 0.0% 0.6%	n=225 98.2% 1.8% 0.0%	.12
**It is the responsibility of the patients to inquire about the cost of care.** **Agree** **Neutral** **Disagree**	n=389 28.2% 29.0% 42.7%	n=161 50.9% 40.4% 8.7%	n=225 12.0% 20.1% 67.1%	.<0001
**It is the responsibility of the physician to discuss issues of costs of care with their patients.** **Agree** **Neutral** **Disagree**	n=386 81.3% 15.0% 3.6%	n=160 84.4% 13.8% 1.9%	n=226 79.2% 15.9% 4.9%	0.23

^a^ p-value compares Harvard Medical School vs. University of Toronto

Table 3 illustrates attitudes among students with respect to specific types of clinical decisions. Seventy-nine percent of both student groups agreed that a blood test should not be ordered unless it has the potential to change management. Wider differences were found in opinions about generic drugs, with 87% of Harvard students agreeing generic drugs should always be considered before brand-name medications, compared with 61% of University of Toronto students who agreed with this statement ( *p* < 0.0001). There were also differences between student groups in perceptions of whether being cost-conscious leads to rationing of healthcare: 30% of Harvard Medical School students endorsed this statement compared with 51% of University of Toronto students who agreed ( *p* < 0.0001). Despite concerns regarding rationing, 80% of students from both countries did not feel it was unethical to factor costs into clinical decision-making.

**Table 3.** Attitudes about cost-conscious decision-making

	Combined	Harvard Medical School	University of Toronto	*p ^a^*
**A blood test should not be ordered unless it has the potential to change management.** **Agree** **Neutral** **Disagree**	n=385 79.2% 11.4% 9.3%	n=159 81.8% 6.3% 11.9%	n=226 77.4% 15.0% 7.5%	.01
**Generic drugs should always be considered before brand-name medications.** **Agree** **Neutral** **Disagree**	n=385 71.9% 20.2% 7.8%	n=160 86.9% 11.3% 1.9%	n=225 61.3% 26.7% 12.0%	<.0001
**Being cost-conscious in the medical field leads to rationing of healthcare.** **Agree** **Neutral** **Disagree**	n=382 42.1% 21.2% 36.6%	n=160 30.0% 18.8% 51.2%	n=222 50.9% 23.0% 26.1%	<.0001
**It is unethical to incorporate issues about cost into decisions about medical care.** **Agree** **Neutral** **Disagree**	n=383 9.1% 14.1% 76.8%	n=160 8.1% 9.3% 82.5%	n=223 9.9% 17% 72.6%	.05

^a^ p-value compares Harvard Medical School vs. University of Toronto

The largest variation between student groups was found in sources of healthcare cost knowledge. Harvard medical students cited an array of sources of knowledge. In contrast, most of the University of Toronto students felt their knowledge came from medical school curricula (61%) ( Table 4). Notably, 85% of students from both schools felt that they had received too little medical school training on costs of care and cost-conscious decision-making and wished that their school would include more formal teaching. Ninety-one percent of students felt that this knowledge was important to their future career as physicians ( Table 5).

**Table 4.** Sources of healthcare cost knowledge

	Combined	Harvard Medical School	University of Toronto	*p ^a^*
**Most of what I know about costs of care comes from medical school.** **Agree** **Neutral** **Disagree**	n=388 50.0% 32.7% 17.3%	n=165 35.1% 53.3% 11.5%	n=223 61.0% 17.5% 21.5%	<.0001
**Most of what I know about costs of care comes from courses other than those I took in medical school.** **Agree** **Neutral** **Disagree**	n=384 20.3% 24.2% 55.5%	n=161 32.9% 30.4% 36.6%	n=223 11.2% 19.7% 69.1%	<.0001
**Most of what I know about costs of care comes from work or volunteering that I did before or during medical school (excluding clinical rotations).** **Agree** **Neutral** **Disagree**	n=385 19.7% 23.9% 56.4%	n=163 28.8% 36.2% 35.0%	n=222 13.1% 14.9% 72.1%	<.0001
**Most of what I know about costs of care comes from personal experience being sick or having a family member of friend who was sick.** **Agree** **Neutral** **Disagree**	n=386 26.4% 21.8% 51.8%	n=164 42.0% 29.8% 28.0%	n=222 14.9% 15.8% 69.4%	<.0001
**Most of what I know about costs of care comes from studying about it on my own.** **Agree** **Neutral** **Disagree**	n=387 28.9% 29.2% 41.9%	n=165 38.8% 43.6% 17.6%	n=222 21.6% 18.5% 59.9%	<.0001

^a^ p-value compares Harvard Medical School vs. University of Toronto

**Table 5.** Attitudes towards cost-conscious curricula

	Combined	Harvard Medical School	University of Toronto	*p ^a^*
**How much formal teaching have you had in medical school so far about costs of care or cost-conscious decision-making?** **Too little** **Just right** **Too much**	n=375 84.8% 9.1% 5.9%	n=155 85.8% 0.0% 14.2%	n=220 81.1% 15.5% 0.4%	<0.0001
**I wish my medical school curriculum would include more formal teaching on costs of care and cost-conscious decision making** **Agree** **Neutral** **Disagree**	n=376 82.5% 14.1% 3.46%	n=155 96.8% 0.7% 2.6%	n=221 72.4% 23.5% 4.1%	<0.0001
**It is important to my future career as a physician to learn about costs of care and how to make cost-conscious decisions during medical school.** **Agree** **Neutral** **Disagree**	n=376 91.0% 6.7% 1.8%	n=155 88.4% 8.4% 3.2%	n=221 92.8% 5.4% 2.4%	<0.0001

^a^ p-value compares Harvard Medical School vs. University of Toronto

At the end of the survey, 42 students from Harvard and 41 students from Toronto contributed with comments and ideas and proposed changes to the graduate medical education (Appendix). Several students commented that cost education should not comprise a separate course, but should be routinely incorporated into existing learning opportunities such as case discussions. One student remarked, “I felt truly incompetent when I started my clinical rotations and realized that I was making decisions.. with complete disregard to patients’ payment ability.. One patient asked me how much a test costs and I had no idea, nor did anyone I asked knew, and we had a really difficult time finding out after hours of searching.”

## Discussion

To our knowledge, this is the first survey of attitudes regarding cost-conscious decision-making between medical students that are learning in two different health system contexts.

The two student populations disagreed on four statements: whether cost-conscious decision-making should be taught in the pre-clinical years, whether patients are responsible for costs of care, the use of brand-name medications, and whether cost-consciousness leads to rationing.

The higher percentage of Canadian students who felt that cost-conscious decision-making should be taught in first- and second-year medical school may reflect the larger proportion of second-year students in the Canadian sample. It is possible that second-year students are interested in learning about the costs of care during their current studies, whereas fourth-year students may view the information as less relevant during pre-clinical years. This also may reflect preclinical resource stewardship curricula at the University of Toronto, awareness of the newly launched Choosing Wisely Canada campaign, or an awareness of the importance of learning cost-consciousness early in clinical training by medical students in a publically funded health system.

The student groups’ differing perspective on patient responsibility likely reflects the payment model in each country. Canadian patients are rarely required to consider costs at the point of service compared to American patients who commonly face out-of-pocket expenses.

While the majority of students agreed that generic drugs should be considered before brand-name medications, significantly more Americans agreed with this statement. Since in Canada out-of-hospital prescriptions are not covered by public health insurance, one might expect more congruence between the two groups. This may reflect an assumption by Canadian medical students that patients will have access to brand-name medications through private insurance. This may also reflect an overall unfamiliarity with financial costs to patients on the part of Canadian medical students.

It is also interesting that there is a split between students on whether or not being cost-conscious leads to rationing of healthcare: half of Canadian students agree that cost-consciousness can result in rationing, while half of Americans disagree with this statement, and around 20% of both groups are neutral with regards to this statement. Practicing in a publically-funded system, Canadian students may be more comfortable with rationing as a resource stewardship tool, while U.S. students may perceive rationing with more stigma. This hypothesis is supported by both groups agreement that it is not unethical to be cost-conscious. Half of Canadian students felt that cost-consciousness can lead to rationing, but only 10% felt that cost-consciousness was unethical; it follows that a portion of Canadian students must not consider rationing unethical.

Overall, medical students in our study expressed a strong desire to learn more about costs of care and cost-conscious decision-making, regardless of the healthcare system funding structure. The majority of students emphasized the importance of having a strong knowledge foundation on this topic, and agreed that physicians in training, attendings, and even medical students in their clinical years should be comfortable addressing issues of cost in the clinical setting. Both Harvard and University of Toronto respondents agreed that it is the physician’s responsibility to discuss costs of care. Both groups of students agreed that blood tests should only be ordered if they may change management.

Several existing resources exist for teaching about cost-conscious care, including the US and Canadian versions of Choosing Wisely and the Alliance for Academic Internal Medicine and American College of Physicians’ Curriculum for High-Value, Cost-Conscious Care. Although these are currently developed for resident and attending physicians, these resources can be adapted for medical students, and our survey can help to inform the areas of similarity and differences that can make the materials relevant for these two healthcare settings. Importantly, teaching about high-value, cost-conscious care is not simply about learning the costs of tests and treatments. Education in this area encompasses critical thinking about the value of a particular test, risks and benefits or tests and treatments, patient preferences for care, and communication with patients about the meaning, pros and cons of different options for care. Thus, efforts to integrate high-value, cost-conscious care can also support more generalized teaching about excellent, patient-centered care.

Our study must be interpreted in the context of our study design. Although all students were surveyed anonymously and questions were designed to minimize social desirability bias, some element of social bias may have persisted due to the controversial nature of this topic. Moreover, because applicable and pre-validated survey questions were not available, questions were developed de novo and may have been subject to interpretation bias. Finally, the generalizability of our study may be limited as Harvard and Toronto students may not reflect the views of all American and Canadian students.

## Conclusion

Our findings support calls for additional health policy training in medical school. ( Patel, Davis and Lypson, 2012) Both our institutions have existing health policy curricula and many students who have completed previous relevant graduate work; however our students still perceive the need for specific training on cost-consciousness to be unmet. This study emphasizes that we may be underestimating the need for education on costs of care.

We were encouraged by students’ attitudes towards the need for a change to the medical curriculum. Not only did the majority of students agree that costs of care should become a necessary part of the medical education, but as the comments in the Appendix reveal, they contributed with practical ideas of how to best introduce this topic and integrate it with the rest of the curriculum. These student attitudes stand in contrast to those of many physicians in practice who are reluctant to assume major responsibility for healthcare costs. Student interest in cost-conscious decision-making may reflect a new appreciation of physician responsibility for growing healthcare costs.

## Take Home Messages

• Medical students at both Harvard Medical School and the University of Toronto valued cost-conscious health care decision making;
• 96% of students agreed that clinicians should be familiar with cost-conscious decision making;
• 80% of students agreed physicians are responsible for discussing healthcare costs with patients;
• 85% of students would like more formal teaching on cost consciousness;
• Despite different health care funding models, students at the two institutions only disagreed on whether cost-conscious decision-making should be taught in the pre-clinical years, whether patients are responsible for costs of care, the use of brand-name; medications, and whether cost-consciousness leads to rationing.

## Notes On Contributors

**Dr. Leon-Carlyle** is a Psychiatry Resident at the University of Toronto, Toronto, ON.

**Dr. McQuillan** is a Staff Nephrologist and Assistant Professor of Medicine at the University of Toronto, Toronto, ON.

**Dr. Baiu** is a General Surgery Resident at Stanford University, Stanford, CA.

**Dr. Sullivan** is an Assistant Professor of Medicine, Beth Israel Deaconess Medical Center and Harvard Medical School, Boston, MA.

**Dr. Dukhovny** is an Associate Professor of Pediatrics, Department of Pediatrics, Oregon Health & Science University, Portland, OR.

**Dr. Shah** is an Assistant Professor of Obstetrics, Gynecology, and Reproductive Biology, Beth Israel Deaconess Medical Center and Harvard Medical School, Boston, MA.

## Appendices

**Appendix: Major Themes and Example Comments:**

Theme	Example Comments
Need for more education	*“I think there are a couple reasons to be informed about costs: 1) Patients will be thinking about it so we need to be able to answer their questions, 2) When there are multiple ways of doing something and one is much less costly than another, it makes sense to choose the less costly one when there won’t be too much harm to the patient. What I am wary of, though, is the idea of factoring in costs to try to hedge bets about whether a particular procedure is needed when there is a fair possibility that doing it would give us valuable information. From a global standpoint it’s impossible to not ration healthcare, but these decisions can be hard on the individual level. I do think that we should learn about how much different things cost as we talk about them in class, and also how we could make those same things less expensive (for those who are interested in research/healthcare delivery optimization/just contributing ideas to the conversation.)”*
	*“It is such an important topic, we really don’t give it any attention. I felt truly incompetent when I started my clinical rotations and realized that I was making decisions for what tests to send with complete disregard to patients’ payment ability or insurance status. One patient asked me how much a test costs and I had no idea, nor did anyone I ask know, and we had a really difficult time finding out after hours of searching. We really should get at least the very basics, medicine has changed so much over the years that this is just something we need to get used to being part of our job...”*
Specific curricular changes	*“During pathophysiology, we should learn about context-dependent use. That is, when would it change management to order a test? We should also be taught to avoid ordering tests for “academic purposes” unless there is a significant weighing of the pros and cons, centering on the patient’s well-being (physical, psychological, financial). We should regularly visit the Choosing Widely guidelines during clinical training. We should be aware of cost variations between the clinical sites at which we train.”*
	*“I think this course should be a part of third year, including in each elective. If you try to do it during 1st or second- year, things will not stick becuase the procedure or test being ‘paid for’ has no context or clinical significance. However, if in the rotation where you are recommending ordering certain tests you learn how much they cost and incorporate that into discussions on treatment plans, it will help the information to stick and become useful.”*
	*“Integrate with Dx and Tx algorithms we learn. Electronic applications or resources with specific costs of specific interventions, repeated to us at multiple points in learning is much better than one discrete class when we forget the information shortly after. I think it’s reasonable to know the average prices of things asked in this survey just as importantly as knowing the medical risks / benefits.”*
	*“Should be integrated into tutorial, especially since so much of the tutorial courses are spent “working up” diagnoses. Many of the tutorial discussions involve rounding up the greatest number of tests possible, with little consideration of ranking the diagnostic tests in order of importance. Should also be essential 3rd year when we write up an Assessment and Plan”*
	*“Perhaps the ideal scenario for 1st and second- year medical students would be to integrate discussions regarding costs of care and cost-conscious decision making into each course while also offering an elective course that focuses solely on the subject. During 3rd and fourth- year there should be a mandatory course that continues and deepens the conversation.”*
Integrating costs with medical education	*“At Harvard, we go through a lot of patient cases. Why not conclude the cases with information on the estimated cost of their care with and without insurance?* *Most easily applicable in the clinical setting, but I think that attendings and residents are unfamiliar with these concepts as well so it would be difficult for them to teach a medical student about costs of care.”*
	*“Have a sum of “costs of care” show up in real-time as one is putting in orders into the hospital computer.”*
	*“I think that cost of care discussions should occur at decision points - ie when students are taught when to perform which action, the costs of each option should be integrated.”*
	*“Healthcare policy course should include more specifics on costs of care and drivers of those costs as well as comparative costs of care (internationally and between domestic providers).”*
	*“At some point in the clinical years, students should be expected to follow some patients (inpt and outpt) through en entire episode of care and understand what happens from the pt’s and family’s perspective, including considerations of cost of care.”*
	*“Having formal didactics about common clinical scenarios (daily labs while inpatient, diagnostic tests for common diseases) would be helpful, especially in 3rd/fourth- year. However, residents also must be taught about costs of care. For example, during my inpatient rotations, my residents insisted on daily labs for everyone regardless if it would change management or be cost-effective.”*
