# Supplementary material for: Assessing Student Attitudes Regarding Cost-Consciousness in Medical Education
Source: MedEdPublish (2016). 2019 Jan 11;8:12. [Version 1] doi: 10.15694/mep.2019.000012.1 (PMC10712531; doi:10.15694/mep.2019.000012.1)
Supplement: Peer review history

# Reviewer report: Reviewer response for version 1

**DOI:** 10.21956/mep.19752.r29633

**Published:** 2019-07-23

**Authors:** Gary D. Rogers

**Affiliations:** 1 Griffith University

This review has been migrated. The reviewer awarded 4 stars out of 5 Thank you, colleagues, for sharing this interesting study comparing medical student experiences and beliefs in relation to cost containment at one US and one Canadian medical school. The authors acknowledge that this is a ‘first step’ investigation in an understudied but very important area. It opens up what is likely to be fruitful avenues for further inquiry, both in relation to how these issues are viewed in a wider range of cultural and economic settings and for the use of qualitative methods to look in more detail at the meaning of the ideas for learners to inform the development of curriculum. There is no doubt that the ethical principle of ‘justice’ requires that considerations of optimal use of scarce resources need to be included in medical curricula going forward and the authors are to be congratulated for beginning to provide an evidence base on how this might best be done.

Reviewer Expertise:

NA

No decision status is available

# Reviewer report: Reviewer response for version 1

**DOI:** 10.21956/mep.19752.r29637

**Published:** 2019-04-30

**Authors:** Saee Deshpande

**Affiliations:** 1 Maharashtra University of Health Sciences, Nashik- VSPM Dental College Nagpur

This review has been migrated. The reviewer awarded 4 stars out of 5 Shared decision making is very necessary wherein patient is partner in the process and it is responsibility of medical professional especially in training years to be cost conscious. In this paper it is very well discussed. However, we should take cognizance of other patient related factors too. Therefore taking a survey is the first step and I request authors to also implement appropriate interventions in this regard in the curriculum to see a positive change in practice.

Reviewer Expertise:

NA

No decision status is available

# Reviewer report: Reviewer response for version 1

**DOI:** 10.21956/mep.19752.r29636

**Published:** 2019-01-18

**Authors:** BALAJI ARUMUGAM

**Affiliations:** 1 TAGORE MEDICAL COLLEGE AND HOSPITAL

This review has been migrated. The reviewer awarded 4 stars out of 5 Congratulations to the entire team of authors involved in this study. The questionnaire including the 25 item scale on the cost consciousness was designed, classified clearly and the outcomes based on the likert scale was expressed in a simpler way. The cost consciousness was classified under 1. Health care responsibility 2. Decision making 3. Source of cost care knowledge 4. Attitude towards cost conscious curriculum As the reviewer of this particular article, I feel that nearly all respondents believed that clinical students (96%), residents (98%), and attending (99%) should be familiar with costs of care and cost-conscious decision-making. This result itself states that there is a need for incorporating the cost conscious component as a part of medical curriculum. The attitude towards cost consciousness curricula was broken into four parts and all were statistically significant. The direction and the dimensions explored in this study among the medical students were nicely explained. Once again congrats to the entire team of authors for publishing this aspect in this forum.

Reviewer Expertise:

NA

No decision status is available

# Reviewer report: Reviewer response for version 1

**DOI:** 10.21956/mep.19752.r29632

**Published:** 2019-01-18

**Authors:** Ariela Marshall

**Affiliations:** 1 Mayo Clinic

This review has been migrated. The reviewer awarded 4 stars out of 5 This is an excellent paper which is extremely well-written and explores a novel area of cost-conscious care, namely medical student perceptions of the adequacy of curricula in this area at their institutions and the importance they place on this topic. The study was well-designed, well-executed, and should contribute significantly to the literature in this area. One small comment: It would be nice to have the survey itself available as a supplemental file. For future studies, the authors might consider (1) including a description of any formal curriculum on cost-conscious care at each institution and (2) surveying practicing physicians involved in medical education at the institutions and comparing their responses to those of the students.

Reviewer Expertise:

NA

No decision status is available

# Reviewer report: Reviewer response for version 1

**DOI:** 10.21956/mep.19752.r29635

**Published:** 2019-01-13

**Authors:** Susmita Reddy Karri

**Affiliations:** 1 Topiwala national medical college and BYL nair charitable hospital, mumbai. india

This review has been migrated. The reviewer awarded 4 stars out of 5 This paper focuses on an extremely important topic which is often not addressed in medical schools. Infact, there is a need to sensitize medical students and junior doctors on this topic, as rightly agreed by most of the respondents. It would be interesting to see similar data from the developing/ under-developed countries as well.

Reviewer Expertise:

NA

No decision status is available

# Reviewer report: Reviewer response for version 1

**DOI:** 10.21956/mep.19752.r29634

**Published:** 2019-01-13

**Authors:** P Ravi Shankar

**Affiliations:** 1 American International Medical University

This review has been migrated. The reviewer awarded 4 stars out of 5 I am of the strong belief that medical students and doctors should be aware of the cost of important diagnostic modalities and treatments. When I was an intern I was often asked by patients or their family members about the cost of different laboratory/diagnostic tests and medicines and unfortunately, often I could not answer the same. When I was a student in the late 1980s cost was never formally addressed in medical school. The process of personal (P) drug selection in pharmacology introduces students to the cost of different drugs used for treating common diseases as cost is one of the parameters considered while choosing P-drugs. This exercise serves as a good introduction to students about cost of medicines. I and my colleagues have now been facilitating this exercise for over 15 years. I found the study conducted at Harvard University in the United States and University of Toronto in Canada to be of great interest. I do agree with the respondents that economic considerations and the cost of various treatments and procedures are not adequately addressed in the curriculum. The authors have adequately mentioned the limitations of their study. The response rate was average (less than 50%) which could also impact the generalizability. This study will be of interest to all medical educators who have to address the challenge of incorporating more teaching-learning about cost-consciousness in the medical curricula.

Reviewer Expertise:

NA

No decision status is available
